# Eco-Stoichiometric Characteristics of Rhizosphere and Bulk Soils of *Smilax china* L. along Vertical Zone Spectrum of Fanjing Mountain

**DOI:** 10.3390/ijerph19148693

**Published:** 2022-07-17

**Authors:** Yingying Liu, Wenmin Luo, Ximei Wen, Guiting Mu, Xianliang Wu, Zhenming Zhang

**Affiliations:** 1 Guizhou Institute of Biology, Guiyang 550009, China; liuyingying2019@126.com (Y.L.); luowenmin2006@163.com (W.L.); mugui6925@163.com (G.M.); wuxianliang1995@163.com (X.W.); 2 Guizhou Institute of Mountain Resources, Guiyang 550002, China; wxm220706@163.com

**Keywords:** ecological stoichiometry, rhizosphere and bulk soil, soil nutrients, *Smilax china* L., altitude

## Abstract

To explore the correlations between nutrients and stoichiometric characteristics in the rhizosphere and bulk soils of understory *Smilax china* L. in forest ecosystems at different altitudes and to clarify the rhizosphere effect of understory vegetation in forest ecosystems and its response strategy to altitude, providing a theoretical basis for better forest ecological environment protection and high-quality development in Fanjing Mountain. Understory *Smilax china* L. at four different altitudes were selected, with the differences and influencing factors of carbon (C), nitrogen (N), phosphorus (P) and potassium (K) mass fractions and stoichiometric ratios in their rhizosphere and bulk soils analyzed. The average mass fractions of total C, total N and alkali-hydrolyzed N in the rhizosphere and bulk soils of *Smilax china* L. at different altitudes were 224.43 and 181.55 g·kg^−1^; 9.56 and 6.81 g·kg^−1^; and 648.19 and 600.70 g·kg^−1^, respectively. The rhizosphere effect of *Smilax china* L. was significant at altitudes of 500 m and 1000 m but became not so prominent with the rise of altitude. The C:N ratio in the rhizosphere and bulk soils ranged from 19.51 to 39.75 and the C:P ratio ranged from 225.29 to 543.05. C accumulation is greater than N accumulation in the rhizosphere and bulk soils of *Smilax china* L., and both present P limitation. Based on the comprehensive analysis of the mass fractions and eco-stoichiometric ratios of soil nutrients, the P limitation in Fanjing Mountain forest ecosystem is commonly seen and should be addressed.

## 1. Introduction

Plants have a close interaction with the seedling soil through their root systems. The soil provides nutrients for plants to grow and the interpenetration and secretion of plant roots also shape the soil to a certain extent [1], causing changes in soil pH and nutrients, thereby leading to differences in physicochemical properties between the rhizosphere and the original soil body [2]. The plant rhizosphere is the place where plants and soil exchange materials (H_2_O, C, N, P and many other substances) and energy, and it is also the most biochemically active part [3,4,5,6]. Therefore, rhizosphere soil can be used to more accurately reflect the restriction of plant growth by soil conditions. Clarifying the accumulation and cycling laws of nutrients such as C, N and P in the rhizosphere and bulk soils is of guiding significance for the evaluation and management of soil fertility, quality and health [7,8]. In addition, a study on the differences between rhizosphere and bulk soil properties during plant growth has important theoretical and practical significance for understanding the ecological habits in plant growth.

Eco-stoichiometry is the study of the balance of multiple chemical elements in biological systems [9]. Different eco-stoichiometries may indicate different nutritional constraints. The stoichiometry of rhizosphere soils can help evaluate the relationships between plant species and underground nutrient balances [10]. However, most studies on soil stoichiometry focus on bulk soils [11,12,13,14,15,16]. There have been an appreciable number of studies evaluating the relationships between plants and underground nutrient balances in the rhizosphere soil using stoichiometric ratios. For instance, Zhu et al. (2020) believed that N addition might gradually enhance rhizosphere P limitation [17]; Dai et al. (2018) believed that soil pH significantly affects C, N and P concentrations and stoichiometries in the rhizosphere soil [18]; Carrillo et al. (2017) analyzed the stoichiometries of C:N:P in the rhizosphere soil of eight herbaceous plants in semi-arid grassland and held that N increase in this plant community is most likely to lead to the loss of C [19]. These studies emphasized the nutritional constraints of plants with different species or growth years in a certain ecosystem inferred from soil stoichiometry, but rarely on the variation law of stoichiometric characteristics of the rhizosphere soil of the same species distributed at different altitudes in the same forest ecosystem. The lack of relevant knowledge on the response of rhizosphere soil to altitude will hinder our in-depth understanding and management of forest ecosystems.

Mountain soil can not only change longitudinally due to formation processes but can also change due to changes in many environmental factors along an altitude gradient [1,20]. Njeru et al. (2018) found that the relationship between altitude and either carbon or nitrogen stocks was linear and positive [21]. Tan et al. (2016) found that soil C and N were not related to altitude, whereas soil P increased with altitude [22]. Forest soils of higher altitudes had a significantly lower soil pH and higher soil organic matter (SOM) and nutrient levels (total nitrogen, available nitrogen and phosphorus) than lower altitudes [23]. The total soil organic carbon density on the northern slope of Mount Taibai showed a decreasing trend with the increase of the altitude gradient [24]. On Taibai mountain, the contents of soil organic carbon and total nitrogen increased first and then decreased with the elevation gradient, and the spatial variation of soil total phosphorus content was small. With the increase in altitude, soil C:N and C:P showed a decreasing trend in the broad-leaved forest belt and changed to an increasing trend in the coniferous forest belt; N:P first rises and then falls with the increase in altitude gradient [25]. On Mao’er Mountain, with the increase in altitude, soil C, N, C/P and N/P all increased; soil P increased first and then decreased; and C/N increased first and then remained stable [26]. There are more and more studies on the composition, distribution and changes of soil nutrients in mountain soils at different altitudes, but there are few studies on the effects of altitude on rhizosphere soils. Therefore, it is of great significance to study the effect of altitude on rhizosphere soil nutrients and their stoichiometric characteristics.

The relative height difference of Fanjing Mountain is up to 2000 m, with a concentrated environmental gradient across the zone at the landscape scale. Its geological history and environmental changes are relatively consistent with almost no obstacles to species migration, which can fully diffuse along the altitude gradient and fill the potential niche. Consequently, it is an ideal place to study the altitude adaptability of the same plant [27]. *Smilax china* L., a vine of *Smilax* L. in *Smillacaceae*, mostly grows under forests and in shrubs below the altitude of 2000 m in the wild. Its rhizomes are commonly used as a Chinese medicinal material. Our previous investigation has found that *Smilax china* L. is distributed in the east line of Fanjing Mountain at 500−2000 m, making it an ideal plant to explore the response to altitude. We hypothesize that: (1) at any altitude, nutrient characteristics (C and N) of *Smilax china* L. rhizosphere soil are significantly higher than those of the bulk soil, presenting a prominent rhizosphere effect; (2) the response trends of *Smilax china* L. rhizosphere soil, C, N and stoichiometric ratios to altitude are consistent with those of the bulk soil.

## 2. Materials and Methods

### 2.1. General Situation of the Study Area

Fanjing Mountain is the first peak on the transition slope from the Yunnan–Guizhou Plateau to hills in Western Hunan, serving as the watershed between Wujiang River and Yuanjiang River, and is also the highest main peak of Wuling Mountains. Fanjing Mountain is located at the junction of Jiangkou, Yinjiang and Songtao counties in northeast Guizhou province, adjacent to the east and west boundaries of Sichuan and Hunan provinces, respectively, with the geographical coordinates of 27°31′~28°41′ N and 108°21′~109°17′ E. Its highest peak is Fenghuang Mountain at 2570.15 m above sea level, followed by Jinding Mountain at 2493.14 m above sea level, with a relative height difference of 2000 m. This region is characterized by an East Asian monsoon climate, with an average annual temperature of 6~17 °C, an average temperature of 3.1~5.1 °C in January and 15~27 °C in July, an active accumulated temperature ≥ 10 °C (AAT10) of 1500~5500 °C, average annual precipitation of 1100~2600 mm and an annual average relative humidity > 80%. The vertical zone spectrum can be divided into four climate zones: mid-subtropical zone, north subtropical zone, warm temperate zone and temperate zone, showing typical humid climate characteristics of mid-subtropical monsoon mountains in China. The soil types from 500 m above sea level to 2000 m above sea level are as follows: Xan Udic Fernalisols, Cab Udi Orthic Entisols, and Fec Hydragric Anthrosols. The forest here is dense and well preserved, with a coverage of more than 80%, forming a relatively balanced forest ecosystem.

### 2.2. Plot Design

**Figure 1:**
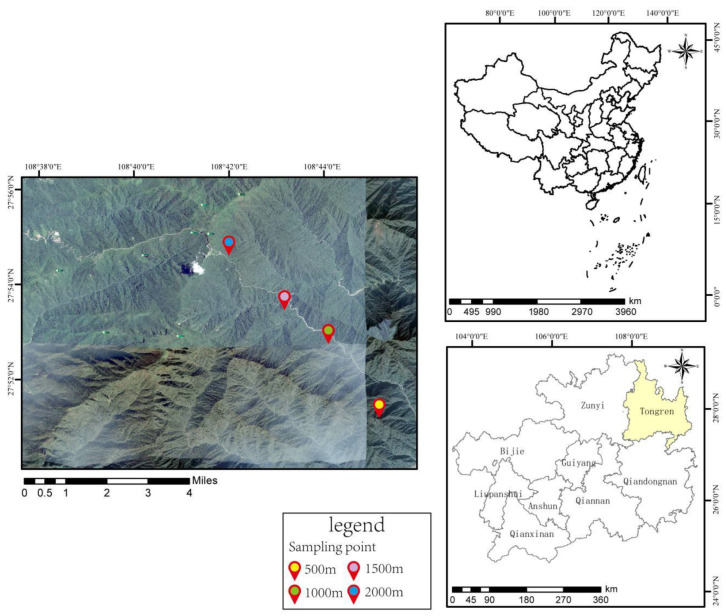
Location of the study area in Mount Fanjing.

According to the growth and distribution of *Smilax china* L. in the study area, in late-June 2021, field investigations were conducted at 500 m, 1000 m, 1500 m, and 2000 m above sea level along the east line of Fanjing Mountain National Nature Reserve. Five different sample points were selected at each altitude, including rhizosphere soil and bulk soil. The location of the study area (Figure 1) and sample plot environment (Table 1) [28] are shown as follows.

### 2.3. Soil Sample Collection

After a full-scale investigation of *Smilax china* L. in the study area, four sample strips at different altitudes (500 m, 1000 m, 1500 m and 2000 m) were selected. Five 30 × 30 m blocks with *Smilax china* L. were established at each altitude. The distance between blocks was above 50 m and each block was divided into 100 patches of 3 × 3 m. The patches with *Smilax china* L., whose ground diameter was 5–8 mm, were numbered. Three seedlings were randomly selected from each patch by lottery for destructive sampling. The rhizosphere or bulk soil from three plants was combined as one repetition. Each sample strip had five repetitions. The roots of *Smilax china* L. were distributed in the topsoil under the forest. The sampling depth is mainly based on the root growth depth, which varied from plant to plant, fluctuating between 0–8 cm and 0–14 cm. The mulch layer was removed before taking the seedlings. Each plant was carefully dug to acquire its whole roots and adhering soils. Referring to the shaking-off method of Riley et al., the bulk soil samples were collected by gently shaking off the soil loosely bound to the root system and plant roots and their closely bound soil (0–4 mm away from the rhizosphere) and bagged as rhizosphere soil samples [29]. Sampling was only conducted after three consecutive sunny days following any rain to ensure an accurate reflection of the soil nutrition and pH.

### 2.4. Determination of Nutrient Concentrations in Soil Samples

The collected soil samples were taken back to the laboratory and placed in a cool and ventilated place for air drying at room temperature, with plant residues and other non-soil substances removed, and dust, acid and base pollution avoided. The air-dried soil samples were ground, sifted through a 2-mm sieve and stored in self-sealing bags for subsequent testing. After treatment with 2.0 mol/L HCl, excess NaOH was added and titrated with calibrated HCl. No inorganic C was found, so it could be determined that the content of TC in the soil samples is equal to organic C. Organic C was measured using the oil bath heating potassium dichromate-volumetric method [30]; Total N was measured using heating digestion-alkaline hydrolysis and diffusion-semimicro Kelvin’s method [31]; Alkaline-hydrolyzed N was measured with alkaline hydrolysis and diffusion-semimicro Kelvin’s method [31]; Total P was measured by heating digestion-anti-molybdenum antimony colorimetry [32]; Available P was measured with anti-molybdenum antimony colorimetry [32]; Total K was measured by heating digestion-flame photometry [33]; Available K was measured using ammonium acetate extraction-flame photometry [33].

### 2.5. Data Processing

The data were statistically analyzed using SPSS25.0 (SPSS Inc., IBM Corporation, Chicago, IL, USA). Before the analysis of variance, the homogeneity of variance of nutrient concentrations and stoichiometric ratios was evaluated using Levene’s test, and most data showed unequal variances. The nonparametric Kruskal–Wallis test was used to analyze the data, and the stepwise reduction method was used for multiple comparisons in the post-test. *p* ≤ 0.05 was considered statistically significant and the data in the figures and tables were expressed as mean ± standard error. The soil nutrients and stoichiometric ratios were mixed and ranked and the rank (i.e., variable RML) was analyzed by two-way ANOVA [34]. Histograms and radar maps were generated using Origin 2019 (OriginLab, Northampton, MA, USA). The rhizosphere effect was expressed as “root/soil ratio” (R/S = rhizosphere soil/bulk soil) [35].

## 3. Results and Analysis

### 3.1. Effects of Rhizosphere Effect (Rhizosphere/Bulk) and Altitude on Soil Nutrients and Stoichiometric Characteristics

As shown in Table 2, rhizosphere effect had no significant effects on TK, AK and soil pH (*p* > 0.05), but a significant effect on AP (*p* < 0.05) and highly significant effects on other soil nutrients and stoichiometric ratios (*p* < 0.01). Altitude presented highly significant effects on SOC, TN, TP, HN, AK, C:K, N:K and pH (*p* < 0.01) and a significant effect on *p*:K (*p* < 0.05). The interaction between altitude and rhizosphere effect showed extremely significant effects on SOC, TN, TP, HN, AK, C:K, N:K and *p*:K (*p* < 0.01) and a significant effect on AP (*p* < 0.05) (Table 2).

### 3.2. Distribution Characteristics of Rhizosphere and Bulk Soil Nutrients of Smilax china L. at Different Altitudes

**Figure 2:**
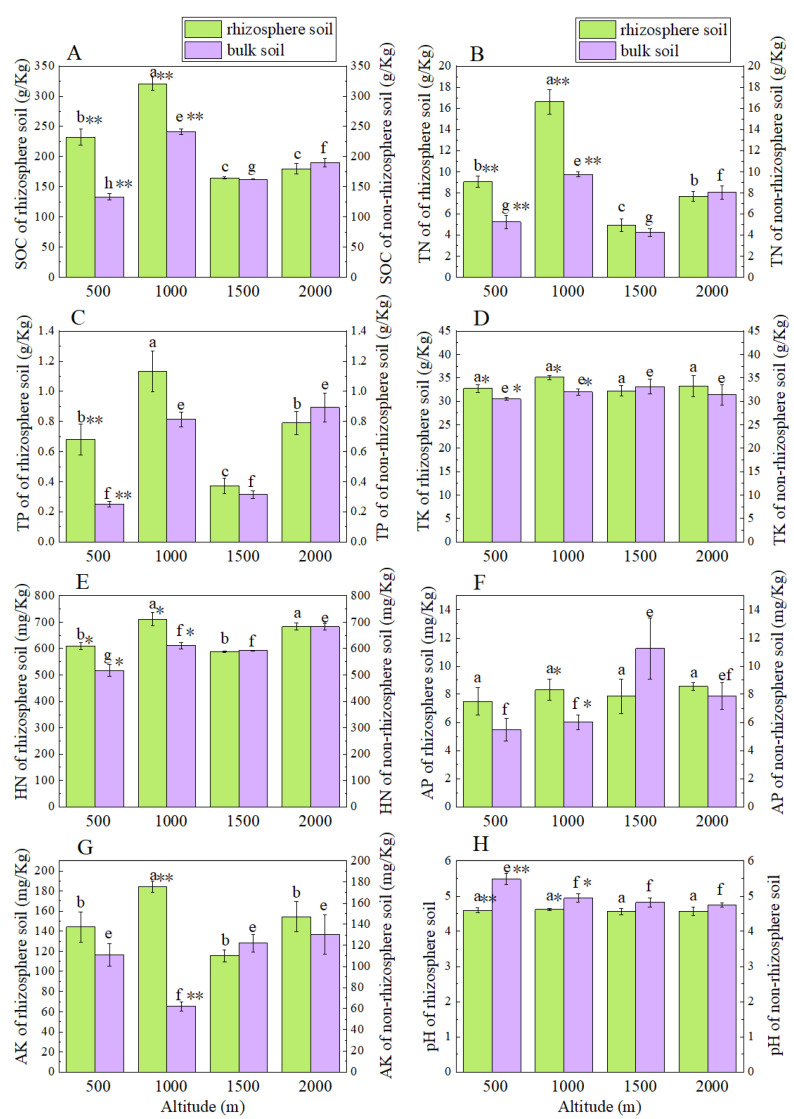
Nutrient contents and pH value in rhizosphere and bulk soils of *Smilax china* L. at different altitudes: (**A**), SOC of rhizosphere and bulk soils; (**B**), TN of of rhizosphere and bulk soils; (**C**), TP of rhizosphere and bulk soils; (**D**), TK of rhizosphere and bulk soils; (**E**), HN of rhizosphere and bulk soils; (**F**), AP of rhizosphere and bulk soils; (**G**), AK of rhizosphere and bulk soils; (**H**), pH of rhizosphere and bulk soils; different lowercase letters (a–c) indicate significant differences at *p* < 0.05 among rhizosphere soils at different altitudes; different lowercase letters (e–h) indicate significant differences at *p* < 0.05 among bulk soils at different altitudes; * and ** indicate significant differences between rhizosphere and bulk soils at the same altitude at *p* < 0.05, *p* < 0.01, respectively.

The organic matter content in the rhizosphere soil of *Smilax china* L. at 500 m and 1000 m above sea level showed enrichment, with highly significant differences (*p* < 0.01). Compared with the bulk soil, the content in the rhizosphere soil was 74.73% and 33.01% higher respectively. However, organic matter content was not significantly different between rhizosphere and bulk soil at 1500 m and 2000 m above sea level (*p* > 0.05). The organic matter contents in the rhizosphere and bulk soils of *Smilax china* L. at 1000 m above sea level were significantly higher than those at 500 m, 1500 m and 2000 m above sea level (*p* < 0.05), by 38.20%, 94.91% and 78.28% in the rhizosphere soil and 81.54%, 48.84% and 27.00% in the bulk soil, respectively (Figure 2A). The rhizosphere soil of *Smilax china* L. at the four altitudes all showed acidification compared with the bulk soil. The pH difference between the rhizosphere and bulk soils of *Smilax china* L. at 500 m altitude was highly significant (*p* < 0.01) (Figure 2H).

TN and HN contents in the rhizosphere soil of *Smilax china* L. at 500 m and 1000 m above sea level showed enrichment. TN in the rhizosphere soil was 72.95% and 70.37% higher than that in the bulk soil, respectively (*p* < 0.01). TN contents in the rhizosphere and bulk soils of *Smilax china* L. at 1000 m above sea level were significantly higher than those at 500 m, 1500 m and 2000 m above sea level (*p* < 0.05) (Figure 2B). Compared with the bulk soil, HN in the rhizosphere soil of *Smilax china* L. at 500 m and 1000 m above sea level was 18.09% and 16.33% higher, with highly significant differences (*p* < 0.05) (Figure 2E).

The changes in p content in the rhizosphere and bulk soils at four altitudes were inconsistent. The total P content in the rhizosphere soil of *Smilax china* L. at an altitude of 1000 m was significantly higher than that at the other three altitudes (*p* < 0.05), by 66.47%, 204.30% and 43.29%, respectively (Figure 2C). There were no significant differences in AP content in the rhizosphere soil at the four altitudes (*p* > 0.05) (Figure 2F).

The changes in K content in the rhizosphere and bulk soils at the four altitudes were inconsistent and TK content showed no significant differences (*p* > 0.05) for the rhizosphere and bulk soils (Figure 2D,G). TK content in the rhizosphere soil of *Smilax china* L. at 500 m and 1000 m above sea level showed significant enrichment (*p* < 0.05) and TK in the rhizosphere soil was 7.43% and 9.79% higher than that in the bulk soil, respectively (Figure 2D).

### 3.3. Stoichiometric Characteristics of Rhizosphere and Bulk Soil Nutrients of Smilax china L. at Different Altitudes

**Figure 3:**
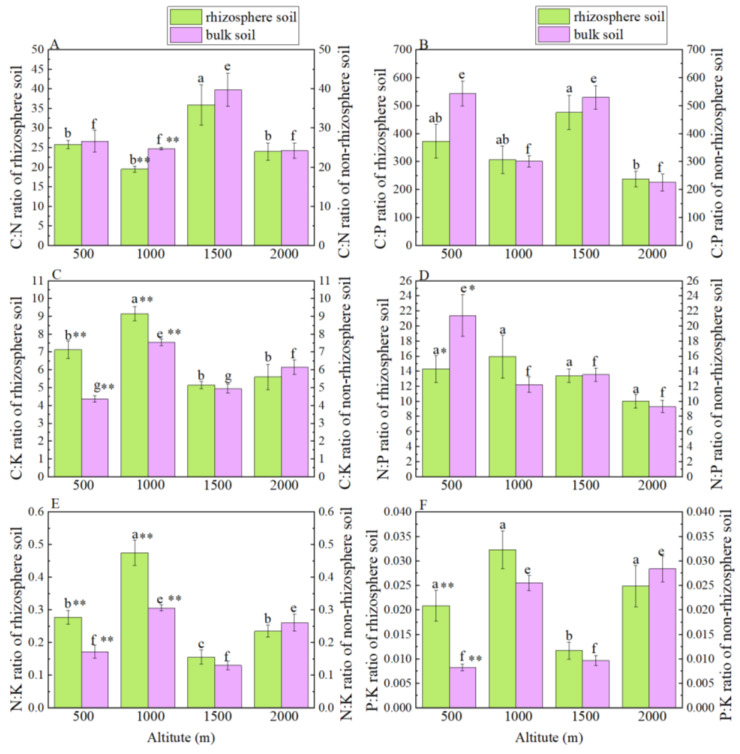
C, N, P and K stoichiometric indexes of rhizosphere and bulk soils of *Smilax china* L. at different altitudes: (**A**), C:N ratio of rhizosphere and bulk soils; (**B**), C:P ratio of of rhizosphere and bulk soils; (**C**), C:K ratio of rhizosphere and bulk soils; (**D**), N:P ratio of rhizosphere and bulk soils; (**E**), N:K ratio of rhizosphere and bulk soils; (**F**), P:K ratio of rhizosphere and bulk soils; different lowercase letters (a–c) indicate significant differences at *p* < 0.05 among rhizosphere soils at different altitudes; different lowercase letters (e–g) indicate significant differences at *p* < 0.05 among bulk soils at different altitudes; * and ** indicate significant differences between rhizosphere and bulk soils at the same altitude at *p* < 0.05, *p* < 0.01, respectively.

The changes in stoichiometric ratios of rhizosphere and bulk soils at the four altitudes were inconsistent. The C:N ratios of rhizosphere and bulk soils at 1500 m above sea level were both the highest and were significantly higher than those at 500 m, 1000 m and 2000 m above sea level (*p* < 0.05) (Figure 3A). The C: P, C: K, N:K and P:K ratios of rhizosphere and bulk soils showed a decreasing–increasing–decreasing trend with the rise of altitude (Figure 3B–F). The C:P ratio in the rhizosphere soil at 1500 m above sea level was the highest and significantly higher than that at 2000 m above sea level (*p* < 0.05) (Figure 3B). The C:K ratio of rhizosphere and bulk soils at 1000 m above sea level was both the largest and significantly higher than that at 500 m, 1000 m and 2000 m above sea level (*p* < 0.05) (Figure 3C). There were no significant differences in the N:P ratio of the rhizosphere soil at different altitudes (*p* > 0.05) (Figure 3D). The N:P ratio of the bulk soil at an altitude of 500 m was significantly higher than that at 1000 m, 1500 m and 2000 m (*p* < 0.05), by 75.21%, 58.10% and 130.13%, respectively (Figure 3D).

### 3.4. Rhizosphere Effects of Soil Nutrients and pH of Smilax china L. at Different Altitudes

**Figure 4:**
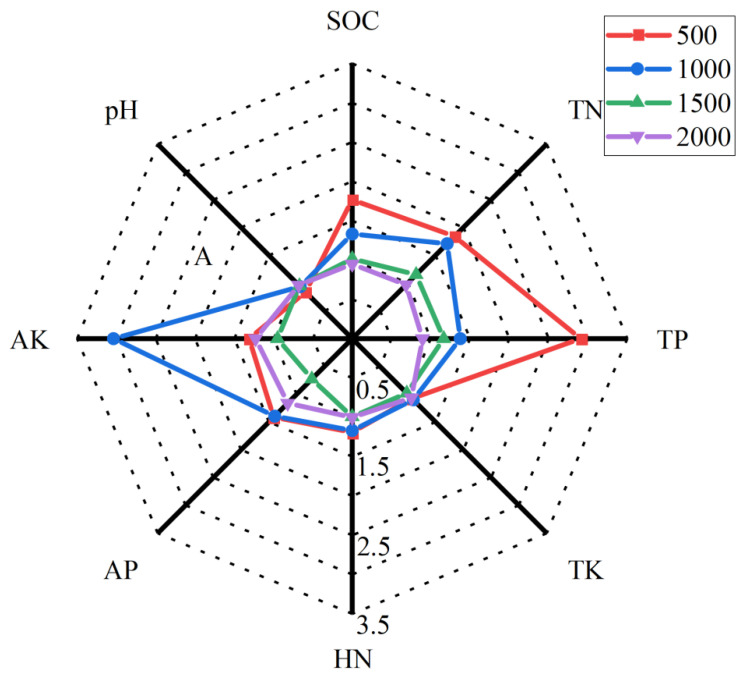
Radar map of rhizosphere effect (R/S = rhizosphere soil/bulk soil).

The R/S values of soil pH were all smaller than 1, presenting a negative effect. The R/S value of soil pH at 500 m above sea level was the lowest (0.84). The R/S values of soil nutrients at 500 m and 1000 m above sea level were >1, indicating a positive effect. The R/S value of TK was the lowest, 1.07 and 1.10, respectively. Except for TK and AK, the R/S value of nutrients at 500 m above sea level were all the highest. Except for AP at 1500 m above sea level, the R/S values of soil nutrients at 1500 m and 2000 m above sea level were close to 1, indicating that rhizosphere effect was not obvious. The R/S value of SOC, TN and TP reduced with the rise in altitude (Figure 4).

### 3.5. Correlation Analysis of Nutrient Characteristics between Rhizosphere or Bulk Soils

**Figure 5:**
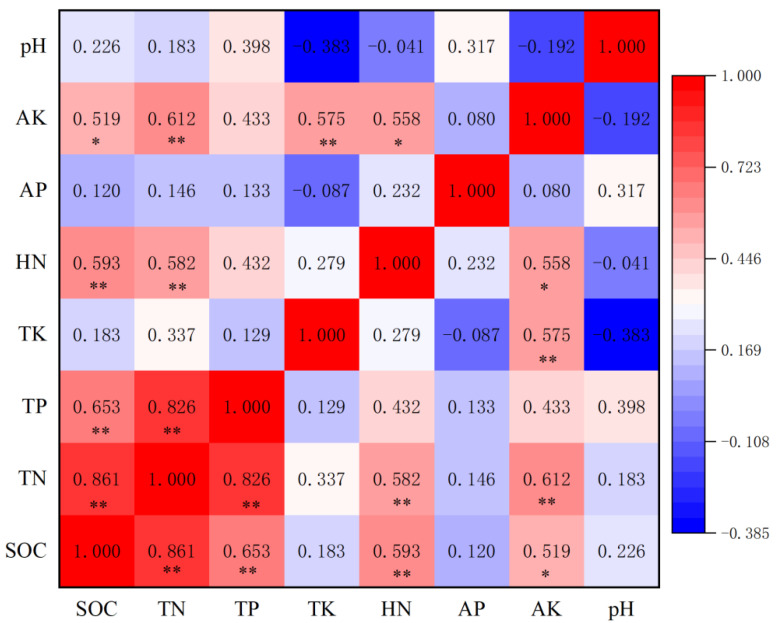
Pearson’s correlation heatmap of rhizosphere soil nutrient concentrations and pH: * Significant at *p* < 0.05; ** significant at *p* < 0.01.

In the rhizosphere soil, SOC had a strong positive correlation with TN, TP and HN (*p* < 0.01) and a significantly positive correlation with AK (*p* < 0.05). There were strong positive correlations between TN and TP, HN and AK (*p* < 0.01), and between TK and AK (*p* < 0.01). HN had a significantly positive correlation with AK (*p* < 0.05) (Figure 5).

**Figure 6:**
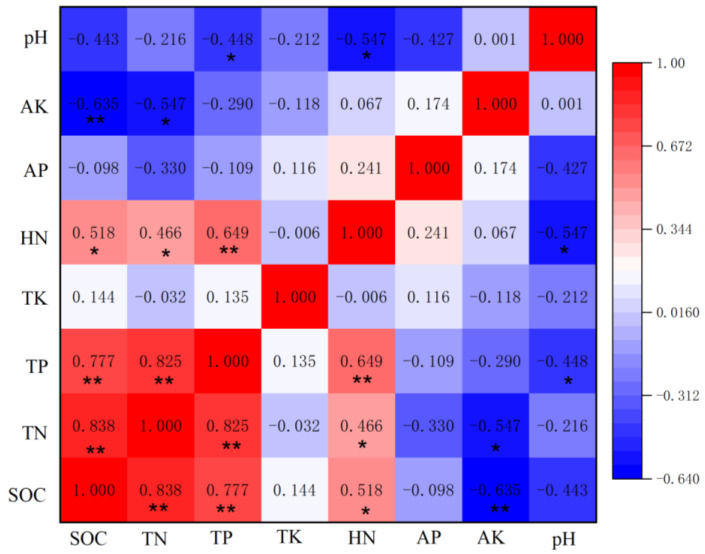
Pearson’s correlation heatmap of bulk soil nutrient concentrations and pH: * Significant at *p* < 0.05; ** significant at *p* < 0.01.

In the bulk soil, SOC had a strong positive correlation with TN, TP and AK (*p* < 0.01) and a significantly positive correlation with HN (*p* < 0.05). TN had a strong positive correlation with TP (*p* < 0.01) and a significantly positive correlation with HN and AK (*p* < 0.05) (Figure 6).

### 3.6. Correlation Analysis of Stoichiometric Characteristics of Nutrients between Rhizosphere or Bulk Soils

**Figure 7:**
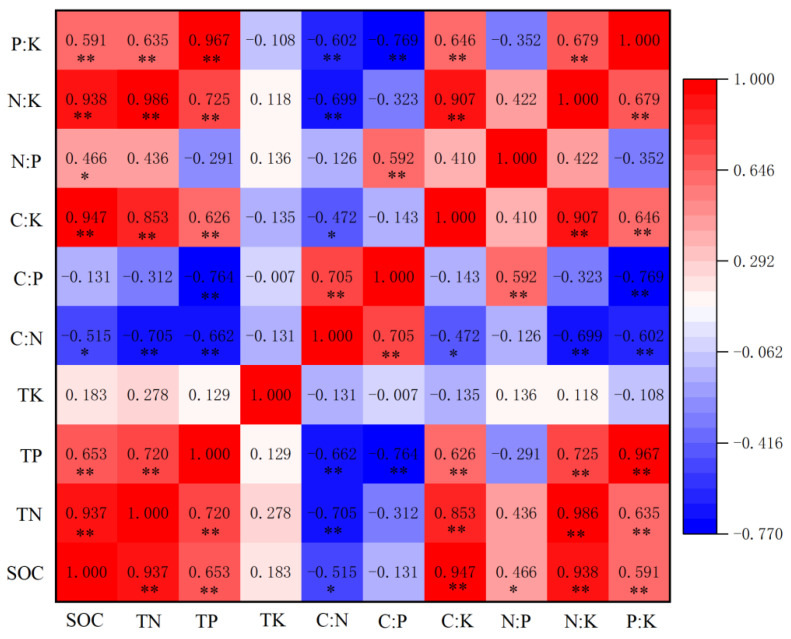
Pearson’s correlation heatmap of rhizosphere soil C, N, P, K concentrations and stoichiometric indexes: * Significant at *p* < 0.05; ** significant at *p* < 0.01.

In the rhizosphere soil, SOC, TN and TP had a strong positive correlation with C:N ratio (*p* < 0.01), and a strong positive correlation with C:K, N:K and P:K ratios (*p* < 0.01). TP had a strong positive correlation with C:P ratio (*p* < 0.01) and SOC had a significantly positive correlation with N:P ratio (*p* < 0.05). C:N ratio had a strong positive correlation with C:P ratio (*p* < 0.01), and a strong negative correlation with N:K and P:K ratios (*p* < 0.01) (Figure 7).

**Figure 8:**
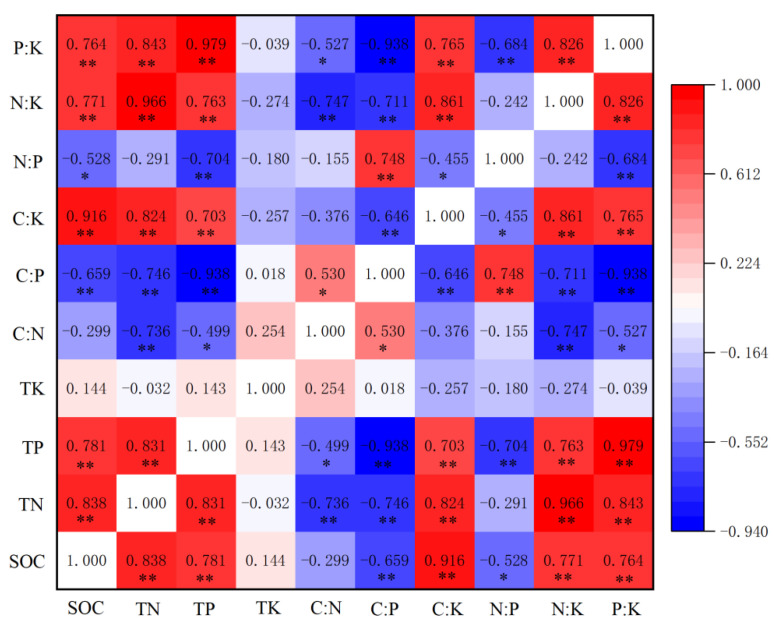
Pearson’s correlation heatmap of bulk soil C, N, P, K concentrations and stoichiometric indexes: * Significant at *p* < 0.05; ** significant at *p* < 0.01.

In the bulk soil, SOC, TN and TP had a strong negative correlation with C:P ratio (*p* < 0.01), and a strong positive correlation with C:K, N:K, and P:K ratios (*p* < 0.01). TN had a strong negative correlation with C:N ratio (*p* < 0.01), TP had a significant negative correlation with C:N ratio (*p* < 0.05), SOC had a significant negative correlation with N:P ratio (*p* < 0.05) and TP had a strong negative correlation with P:K ratio (*p* < 0.01) (Figure 8).

### 3.7. Correlation Analysis of Soil Nutrients and Stoichiometric Characteristics between Rhizosphere and Bulk Soils

**Figure 9:**
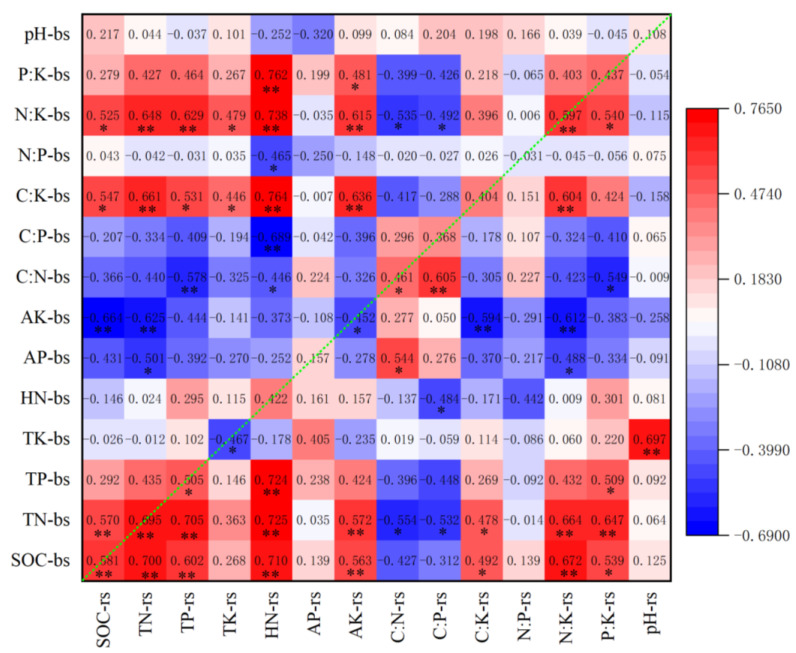
Pearson’s correlation heatmap of bulk soil and rhizosphere soil: * Significant at *p* < 0.05; ** significant at *p* < 0.01; rs, rhizosphere soil; bs, bulk soil.

The correlation trends of SOC, TN, TP, C:K, N:K and P:K in the bulk soil were consistent with those of soil nutrients and stoichiometric ratios in the rhizosphere soil. SOC and TN in the bulk soil had a strong positive correlation with SOC, TN, TP, N:K, HN and AK in the rhizosphere soil (*p* < 0.01) and a significantly positive correlation with C:K (*p* < 0.05). The correlation coefficients between the same indexes of bulk and rhizosphere soils were ranked as TN > N:K > SOC > TP > C:K > P:K > HN > C:K > C:P > AP > pH > N:P > AK > TK in descending order. SOC, TN, TP, C:N and N:K in the bulk soil showed strong positive correlations (*p* < 0.01) or significant positive correlations (*p* < 0.05) with these in the rhizosphere soil, with correlation coefficients of 0.581, 0.695, 0.505, 0.461 and 0.597, respectively (Figure 9).

## 4. Discussion

### 4.1. Effects of Bulk and Rhizosphere Soils on Nutrient Concentrations

In this study, the average mass fractions of total C, total N and alkali-hydrolyzed N in rhizosphere and bulk soils of *Smilax china* L. at different altitudes were much higher than the average levels of terrestrial soil in China, which may be caused by the fact that the humic soil layer of the sampling site is thick, and understory *Smilax china* L. mostly grows in the humic soil and rarely roots in the understory soil layer. The mass fraction of TP in the rhizosphere and bulk soils of *Smilax china* L. at 500 m and 1500 m above sea level were low and relatively poor, consistent with the result of insufficient soil P in subtropical China proposed by Liu et al. (2017) [36]. However, the content of TP in the rhizosphere and bulk soils at 1000 m and 2000 m above sea level was relatively high, which is inconsistent with the argument of insufficient soil P in subtropical China. Rhizosphere soils are generally much richer in nutrients than bulk soils [37]. This is in line with our results that the concentrations of TC, TN, TP and TK in the rhizosphere soil at 500 m and 1000 m above sea level were significantly higher than those in the bulk soil. However, there was no significant rhizosphere nutrient enrichment effect at 1500 m and 2000 m above sea level. It may be related to the natural environmental conditions of Fanjing Mountain. About 11–20% of photosynthetic products in plants are released into the soil in the form of root exudates [38]. The forest ecosystem at an altitude of 1500–2000 m in Fanjing Mountain has large annual precipitation (1400–2000 mm) and a low annual average temperature (8–10 °C). Large precipitation and low temperature may lead to weak photosynthesis and less photosynthetic products of *Smilax china* L., an understory vine, reducing its root exudates secreted into the rhizosphere soil. Therefore, the rhizosphere nutrient enrichment effect of *Smilax china* L. is not significant at these sites.

In addition to the climate, the soil matrix also affects rhizosphere effects according toa recent finding that soils with a finer texture tended to produce a higher level of rhizosphere effects than soils with a coarser texture [39]. The soil textures used by Huo et al. (2017) to analyze the rhizosphere effect were: fine (clay loam, silty loam and loam) and coarse (sandy loam and loamy sand) [39]. In our study, the soil texture at 1500 m is silty loam and the soil texture at the other three altitudes is silty clay loam. Silty clay loam is finer than silty loam. Our results showed that, except for the rhizosphere effect of AP at 1500 m, there was no obvious rhizosphere effect of soil nutrients at 1500 m and 2000 m above sea level, which is not consistent with previous research results [39]. The rhizosphere effect may be affected by soil nutrient availability. Rhizosphere priming may increase the nitrogen supply to plants in a nitrogen-limited system, but it may not occur in a phosphorus-limited system [40]. P limits exist in subtropical forest soils in China [18,41,42]. The P availability of the topsoil at the four altitudes of Mount Fanjing is low, but the rhizosphere effects of soil nutrients at 500 m and 1000 m above sea level were positive effects, which is not consistent with previous research results [40]. Therefore, we speculate that the unobvious rhizosphere effect at 1500 m and 2000 m above sea level does not depend on the initial state of soil but is related to the growth difference of *Smilax China* L. caused by the difference in environmental conditions.

### 4.2. Effects of Bulk and Rhizosphere Soils on C:N:P:K Stoichiometry

The eco-stoichiometric ratios of C, N and P in the rhizosphere soil are important features of ecosystem processes and functions which can be used as effective predictive indexes for the diagnosis of soil nutrient limitation and C, N and P saturation. Rhizosphere soil properties change with different environments [39], thus affecting the soil nutrient cycle and its stoichiometry. In our study, the C:N ratio of rhizosphere and bulk soils first increased and then decreased with the rise in altitude, indicating that the cumulative rate of soil organic C was first higher than N and then lower than N with the rise in altitude. The C:N ratio of the rhizosphere soil ranged from 19.51 to 35.89, and that of the bulk soil ranged from 24.19 to 39.75, higher than the average C:N ratio (12.40) of 0–10 cm soil in the global forest ecosystem, suggesting that the C accumulation is greater than the N accumulation in soil, and the C accumulation rate in the bulk soil is higher than that in the rhizosphere soil [43,44].

Some studies believe that a higher C:P ratio indicates lower P effectiveness [44]. In this study, the C:P ratio of the rhizosphere soil ranged from 237.33 to 475.37, with an average of 348.04, and that of the bulk soil ranged from 225.29 to 543.05, with an average of 399.77, higher than the average C:P ratio (81.90) of 0–10 cm soil in the global forest ecosystem, indicating a lack of P in the rhizosphere and bulk soils of the Fanjing Mountain forest ecosystem. This may be related to the fact that the forest soil of Fanjing Mountain is covered with a thick litter layer, which results in strong enrichment of soil nutrients and a high level of C source available for plant roots to absorb and utilize. The N:P ratio of the rhizosphere soil at the four different altitudes was between 10.03–15.91 and that of the bulk soil ranged from 9.30 to 21.41, much higher than the average N:P ratio (6.60) of 0–10 cm soil in the global forest ecosystem, suggesting that P limitation exists in both rhizosphere soil and bulk soil, which is consistent with previous research results [18,42].

In this study, most roots of *Smilax china* L. grow in the humic soil layer, and a few will extend to deeper soil. Therefore, most of the soil samples used came from the humic soil layer and a small part might come from deeper soil. This may be the main reason why the contents and stoichiometric ratios of C, N and P in the rhizosphere or bulk soil of *Smilax china* L. at different altitudes were significantly different but irregular. These differences may not be the direct result of *Smilax china* L., but due to the entry of roots into different soil depths, which indirectly leads to the different concentrations and stoichiometric ratios of C, N and P in the rhizosphere or bulk soil. The main reasons for the significant but irregular differences in nutrient content and stoichiometric ratios in the rhizosphere or bulk soils of *Smilax china* L. at different altitudes need further study.

### 4.3. Effects of Bulk and Rhizosphere Soils on Stoichiometry

Soil pH changes induced by plant roots determine the biological and chemical availability of mineral nutrients in the rhizosphere environment [45]. Dai et al. held that lower pH is related to more significant N and P nutrient limitations, especially P limitation [18]. Suo et al. (2021) believed that in temperate forests, soil pH is the most important predictor of soil C:N and N:P [46]. In this study, the correlations between rhizosphere soil pH and soil nutrients were not significant. The rhizosphere soil pH had a significantly negative correlation with soil TP and HN, but an insignificant correlation with other indexes, which is not in line with previous research results. In this study, C:N and C:P in the rhizosphere soil showed a highly significant correlation (*p* < 0.01), but only a significant correlation in the bulk soil (*p* < 0.05), indicating that compared with the bulk soil, N and P limitations in the rhizosphere soil are more synergistic, and either nutrient limitation is more likely to cause another limitation, which is similar to the results of previous studies. Cheng et al. (2021) found that the limitation of N and P in rhizosphere soil of a *Pinus. sylvestris* var. mongolica plantation was synergistic [47]; Lin et al. (2021) found that N addition significantly enhanced rhizosphere effects on phosphatase activity in a 60-year-old *Larix kaempferi* plantation [48]. Shan et al. (2018) found that rhizosphere effects responded to interactions of P and N [49]. In this study, C:N, C:P and N:P were only highly affected by the rhizosphere (*p* < 0.01) but had no significant correlations with altitude or the interaction between altitude and rhizosphere (*p* > 0.05). This may be caused by the fact that the roots of *Smilax china* L. entered different soil depths when sampling at different altitudes, which indirectly leads to the irregular changes in the stoichiometric ratios of C, N and P in the rhizosphere or bulk soil.

## 5. Conclusions

The rhizosphere effect is significant at 500 m and 1000 m above sea level but becomes less significant along an elevation gradient. C accumulation is greater than N accumulation in the rhizosphere and bulk soils of *Smilax china* L., and the C accumulation rate in the bulk soil is higher than that in the rhizosphere soil. P limitation exists in both rhizosphere and bulk soils. The level of P limitation in the rhizosphere soil shows no differences among different altitudes, while that in the rhizosphere soil reduces with the increase in altitude. C:N in the rhizosphere and bulk soils is mainly affected by soil total N and presents a significant positive correlation between rhizosphere and bulk soils. C:P in the rhizosphere and bulk soils is mainly influenced by soil total P, but the correlation between rhizosphere and bulk soils is not significant. Based on the comprehensive analysis of the mass fractions and eco-stoichiometric ratios of soil nutrients, the P limitation in the Fanjing Mountain forest ecosystem is commonly seen and should be addressed. The study of the response of *Smilax china* L. rhizosphere and bulk soil nutrients and stoichiometric characteristics to altitude in Fanjing Mountain, a natural forest ecosystem, will not only effectively evaluate the altitude effect of *Smilax china* L. and reveal its eco-stoichiometric response mechanism, but also facilitate our understanding of the rhizosphere effect of understory vegetation in forest ecosystems and its response strategy to altitude.

## Figures and Tables

**Table 1:** Environmental factors of sample plot.

Altitude (m)	500	1000	1500	2000
AR (mm)	1100–1400	1200–1800	1400–2000	1400–2000
MAT (°C)	16.3–16.8	13–15	8–10	8–10
≥10 °C AAAT (°C)	5100–5500	4000–5000	2300–3500	2300–3500
Vegetation types	Evergreen broad-leaved mixed forest	Evergreen broad-leaved forest	Evergreen and deciduous broad-leaved mixed forest	Evergreen and deciduous broad-leaved mixed forest
pH	5.15	4.81	4.78	4.77
SOC (g/kg)	20.64	56.46	93.04	57.12
TN (g/kg)	2.13	3.27	6.34	4.49
TP (g/kg)	0.15	0.27	0.19	0.27
C:N	9.69	17.27	14.68	12.72
C:P	137.60	209.11	489.68	211.56
N:P	14.20	12.11	33.37	16.63
Sand%	26.57	19.52	38.06	32.61
Silt%	52.56	59.98	58.78	50.88
Clay%	20.88	20.50	3.16	16.51
Soil texture	silty clay loam	silty clay loam	silty loam	silty clay loam
Soil types	Xan Udic Fernalisols	Cab Udi Orthic Entisols	Fec Hydragric Anthrosols	Fec Hydragric Anthrosols

Note: AR, Annual rainfall; MAT, Mean annual temperature; ≥10 °C AAAT, ≥10 °C annual active accumulated temperature; SOC, soil organic matter; TN, total nitrogen; TP, total phosphorus; The soil indexes in the above table were all measured in topsoil of 0–20 cm depth in 2014; the data comes from the team’s to-be-published paper ”Evolution of soil texture in mid-subtropical forests in the past 32 years: Taking Fanjing Mountain in Southwest of China as an example”.

**Table 2:** Two-way ANOVA of soil nutrients and stoichiometric ratios (F-value).

Soil Nutrient and Stoichiometric Ratios	Rhizosphere or Bulk Soil	Altitude	Altitude * Soil Type
SOC	59.65 **	40.78 **	27.79 **
TN	54.465 **	16.596 **	5.94 **
TP	25.906 **	8.089 **	6.511 **
TK	0.797	3.577	0.679
HN	30.043 **	11.732 **	6.904 **
AP	3.148 *	1.265	3.088 *
AK	1.097	25.812 **	10.177 **
C:N	14.78 **	3.276	1.546
C:P	20.328 **	1.925	1.741
C:K	18.913 **	12.196 **	8.137 **
N:P	8.467 **	0.01	1.56
N:K	48.976 **	14.298 **	4.973 **
P:K	25.143 **	7.352 *	4.914 **
pH	1.366	23.287 **	1.292

Note: SOC, soil organic matter; TN, total nitrogen; TP, total phosphorus; TK, total potassium; HN, alkali hydrolyzable nitrogen; AP, available phosphorus; AK, available potassium.; * and ** represent significant differences at 5% and 1% levels respectively.

## Data Availability

Data sharing is not applicable to this article.
